# Urinary exosomal long non-coding RNAs as noninvasive biomarkers for diagnosis of bladder cancer by RNA sequencing

**DOI:** 10.3389/fonc.2022.976329

**Published:** 2022-09-01

**Authors:** Bingxian Bian, Li Li, Xing Ke, Hui Chen, Yi Liu, Naisheng Zheng, Yingxia Zheng, Yanhui Ma, Yunlan Zhou, Junyao Yang, Lanshu Xiao, Lisong Shen

**Affiliations:** Department of Clinical Laboratory, Xin Hua Hospital, Shanghai Jiao Tong University School of Medicine, Shanghai, China

**Keywords:** bladder cancer, urinary exosomes, long non-coding RNAs, RNA sequencing, diagnosis

## Abstract

**Introduction:**

Cystoscopy is the standard methodology for diagnosis of bladder cancer (BC), but it is invasive and relatively expensive. Previous studies have found that urinary exosomal long non-coding RNAs (lncRNAs) may act as potential noninvasive biomarkers for diagnosis. Here we identified urinary exosomal lncRNAs that are differentially expressed between BC and controls, and established a panel for diagnosis of BC.

**Methods:**

We performed RNA sequencing in urinary exosomes of 7 controls and 7 patients, subsequently the differentially expressed lncRNAs were detected in training cohort (50 controls and 50 patients) and validation cohort (43 controls and 43 patients). The diagnostic power of lncRNAs for BC was calculated by the area under curve (AUC). The panel for diagnosis of BC was calculated by logistic regression.

**Results:**

The results of RNA sequencing in urinary exosomes showed that 240 upregulated lncRNAs and 275 downregulated lncRNAs were differentially expressed. The levels of MKLN1-AS, TALAM1, TTN-AS1 and UCA1 in BC patients were higher than that in controls in the training and validation cohort by real-time PCR. Using logistic regression, with the combination of these four lncRNAs and NMP22, we identified a panel of five parameters capable of classifying BC patients versus controls on the basis of the training cohort (AUC=0.850). Moreover, the performance of the panel exhibited better performance than either single parameter in the validation cohort.

**Conclusion:**

Collectively, this study confirmed the diagnostic value of lncRNAs for BC by high-throughout urinary exosomal RNA sequencing.

## Introduction

Bladder cancer (BC) is the tenth common diagnosed cancer worldwide (3% of total cases). In man, it is the sixth common diagnosed cancer (4.4% of total cases) and ninth leading cause of cancer death (2.9% of total cases) (1). On average, approximately 70% of bladder cancers (Ta, T1 and CIS) are classified as non-muscle invasive bladder cancer (NMIBC) at diagnosis, and the remainders are defined as muscle invasive bladder cancer (MIBC) (2). In patients of NMIBC at low risk, the 5-year progression free survival rate is 93% (3), whereas in patients of metastatic MIBC, the 5-year relative survival is 5% (distant) to 36% (regional) (4). Therefore, it is important to identify the biomarker for early diagnosis of BC.

Cystoscopy is the standard methodology for diagnosis of BC, but it is invasive and relatively expensive (5). Urine cytology has a sensitivity of 84% in high-grade tumors, but low sensitivity (16%) in low-grade tumors (6). In a recent meta-analysis, the pooled sensitivity and specificity was reported as 0.42 (0.36–0.48) and 1 (0.98–1), respectively (7). Additionally, some biomarkers, such as bladder tumor antigen (BTA), nuclear matrix protein 22 (NMP22) and UroVysion FISH (8–10) are currently commercially available, however wide use of these assays in clinical practice does not seem to have happened due to the lack of diagnostic specificity and sensitivity.

Extracellular vesicles (EVs) can be released by a wide variety of cells as part of their normal physiology and during acquired abnormalities, and exosomes are EVs with a size range of 40 to 160nm in diameter with an endosomal origin (11). Exosomes are initially thought to be a way for cells to excrete waste, however, it has been found that exosomes participate in a variety of physiological and pathological processes such as immune response, antigen presentation, cell differentiation, tumor invasion and so on. Exosomes contain many substances including lipids, nucleic acids and proteins, and the types of nucleic acids include microRNA, rRNA, DNA, lncRNA and so on. Among these bioactive compositions, noncoding RNAs (ncRNAs) are enriched and stable in exosomes, and have drawn much attention about their important roles in cancer development and potential application over the past few years (12). LncRNA is a class of RNA molecules greater than 200nt in length and lacking protein-coding function (13, 14). Moreover, the exosomal membrane can protect lncRNAs from being degraded, and their excellent stability makes exosomal lncRNAs ideal biomarkers for tumor diagnosis (15).

Previous studies found the expression profiles of lncRNAs were significantly different between BC tissues and adjacent normal tissues, which confirmed that lncRNAs could be used as tumor markers for screening BC (16, 17). Duan’s study found that lncRNA was detectable in the serum of BC patients and identified a three-lncRNAs panel for BC diagnosis (18). Beckham’s study confirmed that BC cells released exosomes into urine and lncRNA was stable in exosomes (19). Berrondod’s study found that urinary exosomes from patients with high-grade muscle-invasive urothelial BC (pT2-pT4) disease were enriched in lncRNA HYMA1, LINC00477, LOC100506688 and OTX2-AS1 by RNA-sequencing urinary exosomes from 8 patients and 3 controls, however it lacked the diagnostic performance analysis of these lncRNAs in BC (20). Therefore urinary exosomal lncRNAs showed significant potential as noninvasive biomarkers for diagnosis. Here we identified urinary exosomal lncRNAs that were differentially expressed in BC by RNA sequencing, and established a panel for diagnosis of BC.

## Methods

### Study design and subjects

A total of 100 patients of BC and 100 healthy controls who visited Xin Hua Hospital, Shanghai Jiao Tong University School of Medicine from May 2019 to November 2021 for medical inspection were recruited into this study. Patients with BC were diagnosed pathologically and didn’t undergo surgery, radiotherapy or chemotherapy before urine collection. The staging system to stratify patients of BC is the 2002 UICC TNM classification system, and WHO 2004 grading system is used to classify high-grade *vs* low-grade disease. Controls who received physical examination were selected with similar age and gender proportions to the patients of BC, and they were chosen to ensure they had no history of cancer. All the participants were randomly divided into three cohorts (screening, training and validation cohorts). The study was approved by Xinhua Hospital Ethics Committee. Informed consents were obtained from the participants.

### Urine process

Morning urine of the participants was processed within 4 hours after voiding. We centrifuged urine at 1000rpm for 10 minutes, followed by 2500rpm for 10 minutes to remove any residual debris or bacterial cells. The supernatant was transferred to fresh tubes and stored at -80°C.

### Western blot

Western Blot was performed to verify three markers (HSP70, CD63 and CD81) to confirm successful exosome isolation. Exosomal proteins were isolated according to the instruction of Urine Exosome RNA Isolation Kit (47200, Norgen Biotek, Canada), and 80μl of Cell lysis buffer for Western and IP (P0013, Beyotime, China) and 20μl 5×SDS-PAGE Sample Loading Buffer (P0015, Beyotime, China) were used to resolve exosomal proteins. The mixtures containing exosomal proteins (approximately 20 μl total volume) were heated to 100°C on thermocycler for 5 minutes to fully denature the proteins. The process of western blot was performed according to the standard steps and the PVDF membranes were incubated with primary antibody anti-HSP70 antibody, anti-CD9 antibody and anti-CD81 antibody (EXOAB-KIT-1, SBI, USA) at 4°C overnight and subsequently with goat anti-rabbit IgG H&L secondary antibody (ab175773, Abcam, UK) at room temperature for one hour. The fluorescence detection was performed on the the Li-COR Odyssey Infrared Imaging System (Li-COR Biosciences, USA).

### Transmission electron microscopy

Extracted exosomes were resuspended in 2% PFA, and subsequently 5μl exosome suspension was added to the Cu grid coated with carbon-Formvar. Finally, Cu grid was placed on a 50μl uranium dioxate droplet of ph 7 for 5 minutes and a 50 μl methyl cellulose droplet for 10 minutes. The morphology and size of the exosomes were imaged by the JEM-1230 transmission electron microscope (JEOL, USA).

### Nanoparticle tracking analysis

NTA was used to measure the exosome particle size and concentration using ZetaView PMX 110 (Particle Metrix, Germany) and the software ZetaView 8.04.02. The ZetaView system was calibrated by polystyrene particles (110 nm). Exosome samples were diluted in 1× PBS buffer and analyzed at 11 positions.

### Isolation of RNA

The 50ml supernatant was enriched to 10ml by using Amicon Ultra 15ML 3K Nmwl (UFC900396, Merck Millipore, Germany). Then total RNA was isolated from above 10ml supernatant by using Urine Exosome RNA Isolation Kit according to the manufacturer’s specifications. This kit provided a spin column procedure for the purification of exosomes and the subsequent isolation of exosomal RNA from urine samples.

### RNA sequencing

The ribosomal RNA was firstly removed from the extracted total RNA using the RNase H reagents. After the magnetic beads purified the reaction product, the RNA was fragmented into small pieces by divalent cations for a period of time at appropriate temperature. Subsequently, random primers and reverse transcriptase from the MGIEasy RNA Directional Library Prep Kit were added to the interrupted samples to synthesize the first strand cDNA, and then the second strand cDNA with the dUTP was synthesized by DNA Polymerase I and Rnase H. The cDNA product was subsequently added with a single “A” base and ligated with the adapter. The ligation product was amplified, and the PCR product was thermally denatured into single chains, then a single stranded circular DNA library was obtained by single strand DNA circularization with a bridge primer. The distribution of the library fragments size was detected using the Agilent 2100 bioanalyzer, while the library concentration was quantified using real-time quantitative PCR (TaqMan Probe). The qualified libraries were sequenced on the BGISEQ-500/MGISEQ-2000 System (BGI-Shenzhen, China).

### Reverse transcription-polymerase chain reaction

Total RNA was reverse-transcribed using PrimeScript™ RT reagent Kit (RR037A, Takara, China) in a total volume of 120μl with the following components: 5×PrimeScript Buffer (for Real Time), PrimeScriptRT Enzyme Mix I, Oligo dT Prime (50uM), Random 6 mers (100uM), Total RNA. The RT mixture was incubated at 37°C for 15 minutes, inactivated at 85°C for 5 seconds, and held at 4°C. Real-time PCR was performed using SYBR Premix Ex Taq II (RR820A, Takara, China) in a total volume of 10μl with the following components: SYBR Premix Ex Taq II, PCR forward primer, PCR Reverse Primer, ROX Reference Dye, cDNA and dH_2_O. The real-time PCR was performed at 95°C for 30 seconds, followed by 95°C for 5 seconds, 60°C for 34 seconds and 95°C for 15 seconds, 60°C for 60 seconds. All tests were performed in triplicate.

### Statistical analysis

Comparisons of age were performed by independent t-test and comparisons of gene expressions were performed by non-parametric Mann-Whitney test. Receiver operator characteristic (ROC) curves were generated by changing the thresholds. For each potential threshold, sensitivity, specificity, positive likelihood ratio (PLR) and negative likelihood ratio(NLR) were calculated. Based on the Youden index analysis, we selected the value providing the best tradeoff between sensitivity and specificity. ROC curves were used to evaluate the diagnostic value of single parameter or combinations of different parameters for BC. The diagnostic power was calculated by the area under curve (AUC). The panel of several parameters was calculated by logistic regression. GAPDH was used as housekeeping genes, and 2-ΔΔct was used for relatively quantitation of different expression level. The prognostic value of lncRNAs in BC was analyzed in TCGA by using an online tool GEPIA2. The value of P<0.05 was considered statistically significant. The “limma” package of R (4.1.0, The R Project for Statistical Computing) was employed to calculate the RNA sequencing data. Other statistical analyses were performed using MedCalc 20 (MedCalc Software, Belgium) and GraphPad Prism 9 (GraphPad Software, USA).

## Results

### Baseline characteristics

The study consisted of screening, training and validation cohorts, and clinical parameters were summarized in **Table 1**. There was no significant age and sex difference between controls and patients in three cohorts (all P>0.05). Ta, T1 and CIS were classified as NMIBC, whereas T2,T3 and T4 were classified as MIBC.

**Table 1:** Clinical characteristics of participants in screening, training and validation cohorts.

	Screening cohort			Training cohort			Validation cohort		
	Controls	Patients	P value	Controls	Patients	P value	Controls	Patients	P value
**Age (mean ± SD)**	69.43 ± 10.37	72.00 ± 10.10	0.647	66.54 ± 17.16	65.14 ± 13.94	0.655	70.05 ± 12.85	71.98 ± 9.88	0.437
**Sex**			1.00			1.00			1.00
Male	5	5		42	43		35	36	
Female	2	2		8	7		8	7	
**stage**									
NMIBC		4			34			27	
MIBC		3			16			16	
**grade**									
Low		3			15			9	
High		4			35			34	

NMIBC, non-muscle-invasive bladder cancer. MIBC, muscle-invasive bladder cancer.

### Characterization of exosomes

**Figure 1:**
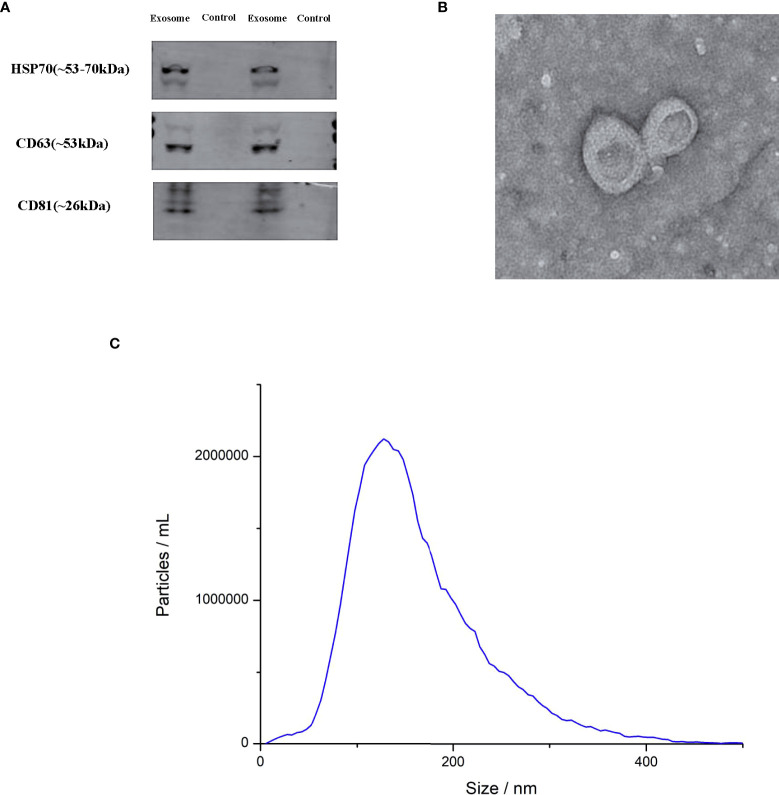
Characterization of exosomes. **(A)** Transmission electron microscopy (TEM) of exosomes **(B)** Western blot for HSP70, CD63 and CD81 in two exosomes and two negative controls **(C)** the exosome particle size and concentration measured by nanoparticle tracking analysis (NTA).

Western blot analysis revealed several typical exosomal markers such as HSP701, CD63 and CD81, which were found in urinary exosomes but not in exosome-depleted urine supernatant (**Figure 1A**). TEM demonstrated an acceptable isolation according to the cup-shape morphology and size range (**Figure 1B**). NTA was performed to detect the size distribution of the exosomes, and the results showed similar size ranges of particles with TEM (**Figure 1C**).

### RNA sequencing

RNA sequencing was performed in urinary exosomes of 7 controls and 7 patients, and a total of 9247 lncRNAs were observed. Among them, 240 upregulated lncRNAs and 275 downregulated lncRNAs were differentially expressed (/log_2_fold change(log_2_FC)/>1). 13 most significantly upregulated lncRNAs (LINC02001, MKLN1-AS, ZBED3-AS1, LINC01612, FLJ22447, HORMAD2-AS1, GRM7-AS3, LOC105371240, DNMBP-AS1, TALAM1, TTN-AS1, UCA1 and ITGA9-AS1) and 13 most significantly downregulated lncRNAs (TP53TG1, INO80B-WBP1, LOC102724902, IDI2-AS1, LOC107985976, LOC105379549, GATA2-AS1, LOC105372310, LOC101929572, LOC105370333, LINC01510, LOC100507412 and CTC-338M12.4) were selected for further analysis. The log_2_FC and primers of these lncRNAs and GAPDH were listed in **Table S1**.

### Real-time PCR

**Figure 2:**
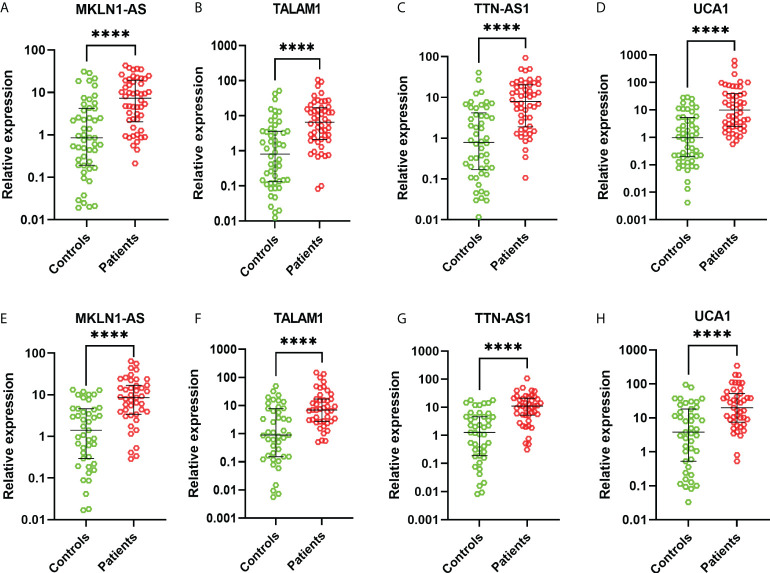
Relative expressions of lncRNAs in the training and validation cohorts. **(A)** MKLN1 in the training cohort **(B)** TALAM1 in the training cohort **(C)** TTN-AS1 in the training cohort **(D)** UCA1 in the training cohort **(E)** MKLN1 in the validation cohort **(F)** TALAM1 in the validation cohort **(G)** TTN-AS1 in the validation cohort **(H)** UCA1 in the validation cohort.

**Figure 3:**
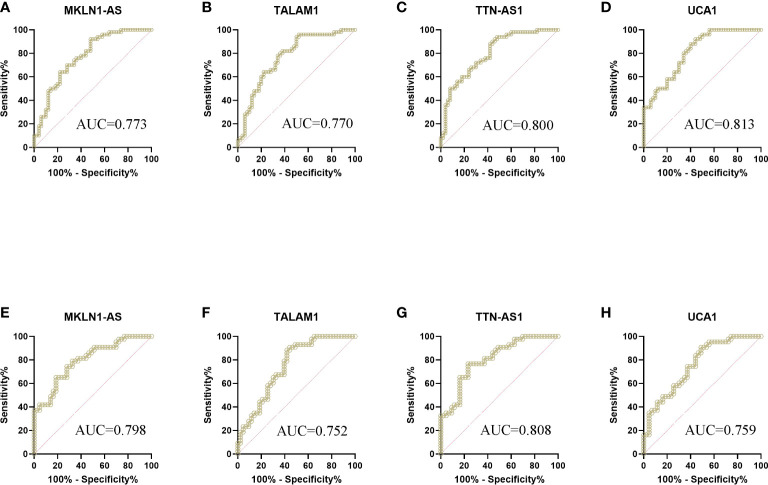
ROC curve analysis of lncRNAs for the diagnosis of bladder cancer from controls. **(A)** MKLN1 in the training cohort, AUC=0.773 **(B)** TALAM1 in the training cohort, AUC=0.770 **(C)** TTN-AS1 in the training cohort, AUC=0.800 **(D)** UCA1 in the training cohort, AUC=0.813 **(E)** MKLN1 in the validation cohort, AUC=0.798 **(F)**TALAM1 in the validation cohort, AUC=0.752 **(G)**TTN-AS1 in the validation cohort, AUC=0.808 **(H)**UCA1 in the validation cohort, AUC=0.759.

These 26 lncRNAs were detected in 23 controls and 23 patients. Only the significantly differentially expressed lncRNAs were selected to be analyzed in another cohort. Then four lncRNAs were further detected in another cohort of 27 controls and 27 patients. The levels of MKLN1-AS, TALAM1, TTN-AS1 and UCA1 in patients were higher than that in controls (all P<0.0001, **Figure 2**). Subsequently, ROC curve was performed to confirm the capacity of these lncRNAs to distinguish patients of BC from controls. For MKLN1-AS, TALAM1, TTN-AS1 and UCA1, the AUC was 0.773 (0.679-0.851), 0.770 (0.675-0.848), 0.800 (0.709-0.874), 0.813(0.723-0.884) with a sensitivity of 92%, 96%, 94%, 92% and a specificity of 52%, 48%, 52%, 56%, respectively (**Figures 3A–D** and **Table S2**).

In the validation, the four lncRNAs were detected for further analysis by real-time PCR in 43 controls and 43 patients. For MKLN1-AS, TALAM1, TTN-AS1 and UCA1, the AUC was 0.798 (0.697-0.877), 0.752 (0.647-0.839), 0.808 (0.709-0.885), 0.759 (0.655-0.845), with a sensitivity of 79.07%, 90.7%,76.74%, 90.7% and a specificity of 67.44%, 55.81%, 76.74%, 90.7%, respectively (**Figures 3E–H** and **Table S3**).

### NMP 22

NMP 22 is an abundant component of the nuclear matrix proteins, which may exist in urine of persons with risk factors or symptoms of BC or with a history of bladder cancer. All participants included in the study were tested for NMP22 as well. The AUC of NMP22 for the diagnosis of BC was 0.650 (0.548-0.743), 0.698 (0.589-0.792) with a sensitivity of 42%, 48.84% and a specificity of 88%, 90.7% in the training and validation cohort, respectively (**Figure S1**).

### Establishment of the panel

**Figure 4:**
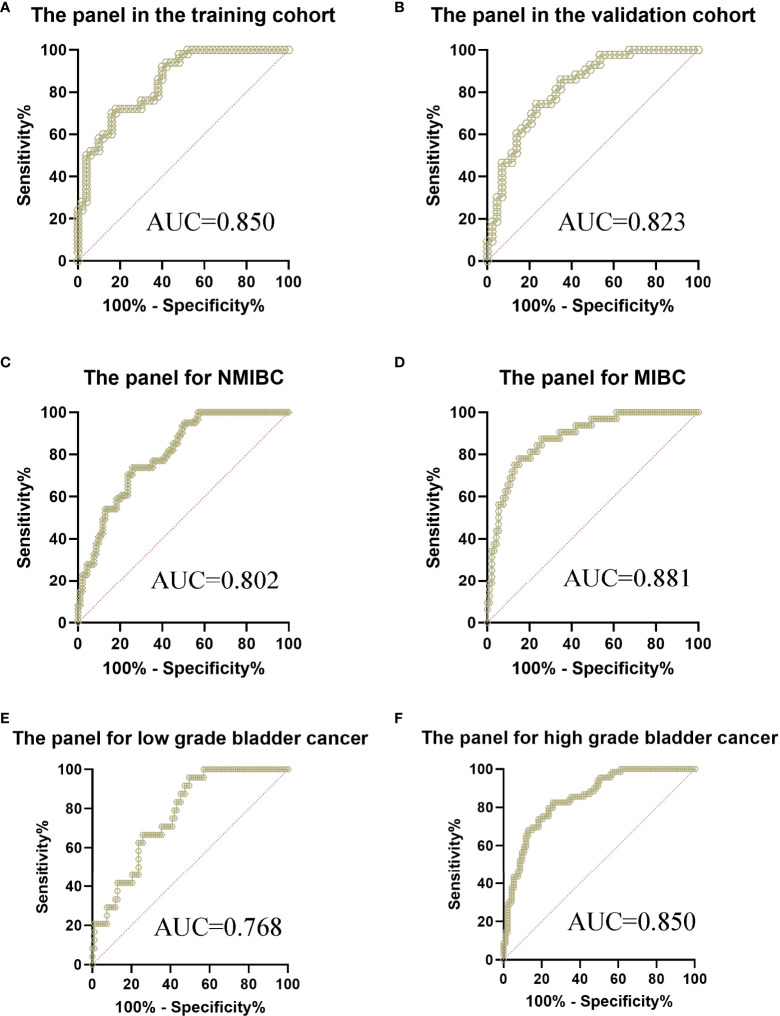
ROC curve analysis of the panel for the diagnosis of bladder cancer from controls. **(A)** The panel in the training cohort, AUC=0.850 **(B)** The panel in the validation cohort, AUC=0.823 **(C)** The panel for the diagnosis of NMIBC from controls, AUC=0.802 **(D)** The panel for the diagnosis of MIBC from controls, AUC=0.881 **(E)** The panel for the diagnosis of low grade bladder cancer from controls, AUC=0.768 **(F)** The panel for the diagnosis of high grade bladder cancer from controls, AUC=0.850.

Using logistic regression, with the combination of these four lncRNAs and NMP22, we identified a panel of five parameters (MKLN1-AS, TALAM1, TTN-AS1, UCA1 and NMP22) capable of classifying patients versus controls on the basis of the training cohort. The predictive probability was calculated using the following equation: logit(P)=-1.0756-0.0837*(MKLN1-AS)+0.0056*(TALAM1)+0.0822*(TTN-AS1)+0.0735*(UCA1)+1.3699*(NMP22). The AUC of the panel was 0.850 (0.764-0.913) with a sensitivity of 72% and a specificity of 82% (**Figure 4A** and **Table S4**), which exhibited better performance than either single parameter.

### Validation of the panel

The parameters extracted from the training set were used to predict the probability of classifying patients versus controls. ROC analysis was performed to evaluate the performance of the panel in the validation cohort. Similarly, the AUC was 0.823 (0.726-0.897) with a sensitivity of 86.05% and a specificity of 65.12% (**Figure 4B**), which exhibited better performance than either single parameter in the validation cohort. Furthermore, we evaluated the performance of the panel in different subgroups of all participants. The AUC of the panel was 0.802 (0.730-0.861) with a sensitivity of 73.77% and a specificity of 74.19% when classifying Ta-T1 patients versus controls (**Figure 4C**). Meanwhile, the AUC of the panel was 0.881 (0.811-0.932) with a sensitivity of 78.12% and a specificity of 84.95% when classifying T2-T4 patients versus controls (**Figure 4D**). Subsequently, the AUC of the panel was 0.768 (0.681-0.841) with a sensitivity of 95.83% and a specificity of 50.54% when classifying low grade patients versus controls (**Figure 4E**). Furthermore, the AUC of the panel was 0.850 (0.786-0.901) with a sensitivity of 82.61% and a specificity of 74.19% when classifying high grade patients versus controls (**Figure 4F**).

### The prognostic value of lncRNAs

**Figure 5:**
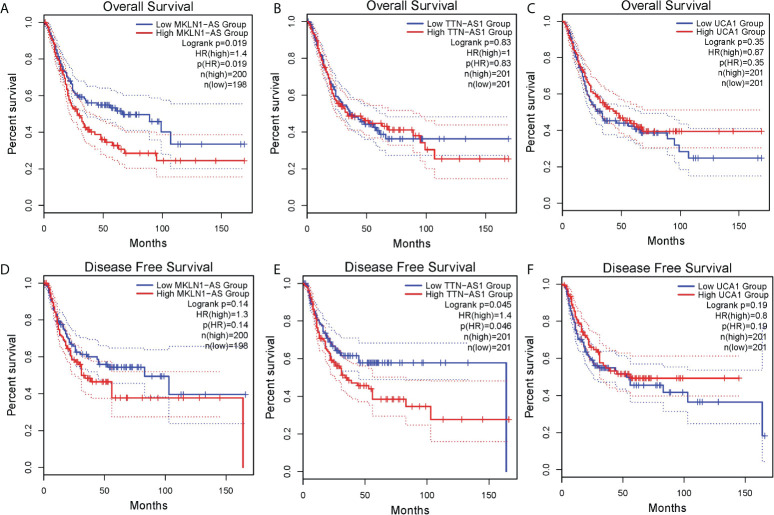
Survival analysis of lncRNAs in bladder cancer was analyzed by using an online tool GEPIA. **(A)** MKLN1-AS for overall survival (OS) **(B)** TTN-AS1 for OS **(C)**UCA1 for OS **(D)** MKLN1-AS for disease free survival(DFS) **(E)** TTN-AS1 for DFS **(F)** UCA1 for DFS.

To further investigate the prognostic value of above lncRNAs, we analyzed related data of TCGA by using an online tool GEPIA2. The results showed high MKLN1-AS group was associated with a poorer overall survival (OS) compared with low MKLN1-AS group (**Figure 5A**). Meanwhile, there was no significant correlation between OS and TTN-AS1 or UCA1 (**Figures 5B** **, C**). Furthermore, high TTN-AS1 group was associated with a poorer DFS (disease free survival) compared with low TTN-AS1 group (**Figure 5E**). Meanwhile, there was no significant correlation between these DFS and MKLN1-AS or UCA1 (**Figure 5D, F**). The TALAM1-related data was not found in the database.

### The correlations between clinical parameters and lncRNAs

The relationships between four lncRNAs and clinical parameters were further explored in the whole cohorts. Significant correlations were found between tumor stage and two lncRNAs (MKLN1-AS and UCA1), whereas no significant correlation was found between tumor stage and TALAM1 or TTN-AS1 (**Table 2**). We next evaluated the relationship between tumor grade and these lncRNAs, and found no significant correlation (all P>0.05). Meanwhile, there was no significant correlation between these five lncRNAs and sex or age.

**Table 2:** The correlations between clinical parameters and lncRNAs.

	Stage			Grade			Age			Sex		
	NMIBC	MIBC	P value	Low	High	P value	≤68	>68	P value	Male	Female	P value
**MKLN1-AS(Median, Interquartile range)**	5.53(1.47-14.64)	9.64(4.5-23.02)	0.036	7.26(1.62-13.81)	8.86(3.12-21.62)	0.229	8.63(3.62-17.15)	6.45(2.04-22.28)	0.712	7.976(2.94-17.53)	9.41(1.42-24.25)	0.591
**TALAM1(Median, Interquartile range)**	7.59(2.43-16.68)	6.54(2.70-17.46)	0.872	7.28(1.89-14.90)	6.61(2.77-17.29)	0.799	7.48(2.87-16.88)	6.05(1.96-17.05)	0.364	6.86(2.55-17.03)	7.10(2.67-15.26)	1
**TTN-AS1(Median, Interquartile range)**	6.96(2.11-21.07)	11.24(5.23-19.75)	0.365	6.61(2.64-16.06)	10.88(3.52-21.23)	0.339	10.19(4.41-19.11)	9.43(2.03-21.75)	0.701	9.99(3.18-20.81)	9.94(2.14-17.87)	0.923
**UCA1(Median, Interquartile range)**	10.17(3.38-37.49)	30.24(8.15-55.18)	0.036	10.49(3.49-38.79)	16.46(4.79-44.95)	0.424	15.82(4.38-45.49)	12.40(3.55-40.35)	0.776	15.19(3.96-39.52)	13.36(4.19-42.63)	0.864

NMIBC, non-muscle-invasive bladder cancer. MIBC, muscle-invasive bladder cancer.

## Discussion

In this study, we performed high-throughout sequencing in the screening cohort and found 240 upregulated lncRNAs and 275 downregulated lncRNAs were differentially expressed. 13 most significantly differentially upregulated lncRNAs and 13 most significantly differentially downregulated lncRNAs were selected for further analysis. Subsequently, we found four lncRNAs (MKLN1-AS, TALAM1, TTN-AS1 and UCA1) were significantly higher in BC than in controls in the training and validation cohort.

Urothelial carcinoma associated 1 (UCA1) was firstly reported in the tissue and urine in 2006 and the results showed that UCA1 was highly specific and sensitive in the diagnosis of BC (21). UCA1 was involved in BC progression through the activation of the oncogenic PI3K-AKT-mTOR pathway, positively regulating glutaminase 2 (GLS2) expression, upregulating expression of high mobility group protein B1 (HMGB1) and downregulating p21 expression (22). Yazarlou et al. found urinary exosomal LINC00355, UCA1-203 and MALAT1 were significantly higher in BC compared to controls, whereas UCA1-201 was significantly decreased, and discovered a panel of these lncRNAs for the diagnosis of BC (23).

Some previous studies also focused on the diagnostic value of urinary exosomal lncRNAs for BC by RNA sequencing. For instance, Huang et al. conducted RNA sequencing of BC tissue, selected differential lncRNAs between tumor tissues and normal tissues for further analysis, and finally demonstrated the urinary exosomal mRNAs and lncRNAs (MIR205HG and GAS5) panel exhibited a good performance in the diagnosis of BC (24). However, to our known, we investigated the diagnostic value of TTN-AS1, MKLN1-AS and TALAM1 for BC by high-throughout urinary exosomal RNA sequencing for the first time.

TTN-AS1 is a lncRNA that binds to titin mRNA and has pro-oncogenic effects in many cancers. Overexpression of TTN-AS1 correlates with poor prognosis in different cancers (25). For instance, TTN-AS1 promoted proliferation and invasion of breast cancer cells by interaction with the miR-139-5p/ZEB1 axis (26). Recent studies reported knocking down TTN-AS1 resulted in inhibiting the ability of proliferation and invasion of BC cells, which supported TTN-AS1 as a biomarker for BC (27). However, the diagnostic role of TTN-AS1 in BC had not been reported.

MKLN1-AS was reported to aggravate hepatocellular carcinoma progression by functioning as a molecular sponge for miR-654-3p, thereby promoting hepatoma-derived growth factor expression (28). In another study, MKLN1-AS intensified proliferation, migration and invasion of hepatocellular carcinoma cells *via* YAP1 (29). However, the diagnostic role of MKLN1-AS in BC had not been reported.

Down-regulation of TALAM1 was shown to greatly impact on the capacity of breast cancer cells to migrate *in vitro* or to populate the lungs of immunocompromised mice (30). TALAM1 is a broadly expressed natural antisense transcript at the MALAT1 locus, and positively regulates MALAT1 levels by promoting the 3’ end cleavage and maturation of MALAT1 (31). A panel consisting of three lncRNAs (MALAT1, PCAT-1 and SPRY4-IT1) which had been reported to play functional roles in tumorigenesis possessed considerable clinical value in the diagnosis (15), whereas the diagnostic role of TALAM1 in BC had not been reported.

NMP 22 Bladder Check Test is based on the detection of a nuclear mitotic apparatus protein which is secreted from dead cells. The AUC of the traditional biomarker was 0.650 with a sensitivity of 42% and a specificity of 88%, which was similar with the results of previous studies (32). Therefore, considering its weak performance, we combined it with four lncRNAs and found a panel of five parameters with good performance (AUC=0.850). The performance of the panel in the validation was similarly with the results in the training cohort.

In the subgroup, the diagnostic value of the panel was still satisfactory for NMIBC (AUC= 0.802), which meant the early diagnosis and a better prognosis of BC. For the MIBC, the diagnostic value of the panel was excellent (AUC=0.881). In addition, we investigated the correlations between clinical parameters and four lncRNAs. We found significant correlations were found between tumor stage and two lncRNAs (MKLN1-AS and UCA1), which indicated the level of two lncRNAs could reflect the cancer severity and play a role in the prognostic value for BC. But when we evaluated the relationship between tumor grade and these lncRNAs, no significant correlation was observed. To some extent, this was due to fewer low grade BC patients included in our study.

There are limitations to this study. Firstly, our study discovered four BC-related lncRNAs. UCA1 was reported to involve in BC progression through a few pathways and act as a diagnostic biomarker for BC. However, MKLN1, TALAM1 and TTN-AS1 was still needed to be studied for the mechanism of involvement in BC progression and the diagnostic value in BC. Secondly, significant correlations were found between tumor stage and two lncRNAs (MKLN1-AS and UCA1), which tentatively revealed the relationship between lncRNAs and the degree of the disease. Further large cohort studies are still needed to confirm the relationship between urinary exosomal lncRNAs and prognosis of BC. Thirdly, this is a single-center study, and further large multi-center studies are needed to evaluate the diagnostic value of urinary exosomal lncRNAs in BC.

## Conclusion

Collectively, the levels of MKLN1-AS, TALAM1, TTN-AS1 and UCA1 in patients were higher than that in controls. Hence we identified a panel consisting of MKLN1-AS, TALAM1, TTN-AS1, UCA1 and NMP22, which exhibited good performance for the diagnosis of BC from controls. Further large cohort studies are necessary to evaluate the prognostic value of these lncRNAs in BC.

## Data availability statement

The original contributions presented in the study are publicly available. This data can be found here: https://www.ncbi.nlm.nih.gov/sra/PRJNA872870.

## Ethics statement

This study was reviewed and approved by The Ethical Committee, Xin Hua Hospital, Shanghai Jiao Tong University School of Medicine. The patients/participants provided their written informed consent to participate in this study.

## Author contributions

BB, LL and XK contributed equally to this work. LS and BB conceived and designed the experiments. LL, XK and BB collected urine samples and the corresponding clinical information. BB, LL, XK, YL, NZ, YM, YLZ and LX performed the experiments. BB, LL, NZ, HC, JY and YXZ analyzed the data. BB, LL and XK wrote the manuscript. LS and BB revised the manuscript. All authors contributed to the article and approved the submitted version.

## Funding

This work was supported by the National Science Foundation of China (81672363, 81873863), the Key Specialty Development Program of Xin Hua Hospital and Shanghai Municipal Health Commission, Clinical Research Plan of SHDC (16CR3057A), Medicine and Engineering Cross Research Foundation of Shanghai Jiao Tong University (YG2017ZD02).

## Conflict of interest

The authors declare that the research was conducted in the absence of any commercial or financial relationships that could be construed as a potential conflict of interest.

## Publisher’s note

All claims expressed in this article are solely those of the authors and do not necessarily represent those of their affiliated organizations, or those of the publisher, the editors and the reviewers. Any product that may be evaluated in this article, or claim that may be made by its manufacturer, is not guaranteed or endorsed by the publisher.

## Supplementary material

Supplementary Figure 1 ROC curve analysis of NMP22 for the diagnosis of bladder cancer from controls. **(A)** NMP22 in the training cohort, AUC=0.650 **(B)** NMP22 in the validation cohort, AUC=0.698.

## Abbreviations

BC, bladder cancer; lncRNA, long non-coding RNA; AUC, area under curve; NMIBC, non-muscle invasive bladder cancer; MIBC, muscle invasive bladder cancer; BTA, bladder tumor antigen; NMP22, nuclear matrix protein 22; EVs, extracellular vesicles; ncRNAs, noncoding RNAs; TEM, transmission electron microscopy; NTA, nanoparticle tracking analysis; RT-PCR, reverse transcription-polymerase chain reaction; ROC, receiver operator characteristic; PLR, positive likelihood ratio; NLR, negative likelihood ratio; FC, fold change; OS, overall survival; DFS, disease free survival; UCA1, urothelial carcinoma associated 1; GLS2, glutaminase 2; HMGB1, high mobility group protein B1.
