# Supplementary figures and images for: Urinary exosomal long non-coding RNAs as noninvasive biomarkers for diagnosis of bladder cancer by RNA sequencing
Source: Front Oncol. 2022 Sep 1;12:976329. doi: 10.3389/fonc.2022.976329 (PMC9477086; doi:10.3389/fonc.2022.976329)

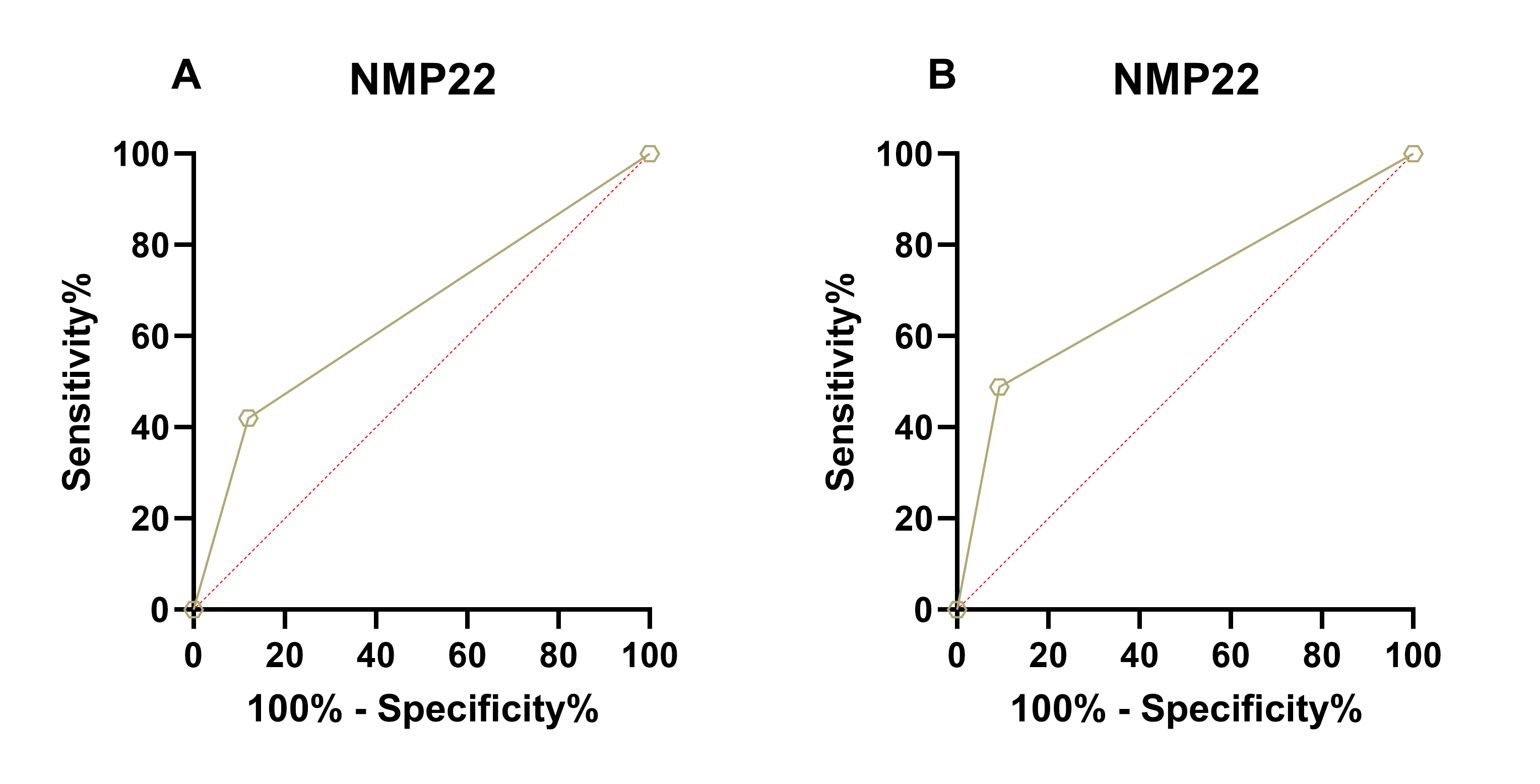

Supplement: Supplementary Figure 1 — ROC curve analysis of NMP22 for the diagnosis of bladder cancer from controls. (A) NMP22 in the training cohort, AUC=0.650 (B) NMP22 in the validation cohort, AUC=0.698. [file Image_1.tif]
