# Supplementary material for: Urinary exosomal long non-coding RNAs as noninvasive biomarkers for diagnosis of bladder cancer by RNA sequencing
Source: Front Oncol. 2022 Sep 1;12:976329. doi: 10.3389/fonc.2022.976329 (PMC9477086; doi:10.3389/fonc.2022.976329)
Supplement: Supplementary file 2 [file Table_1.docx]

| **Table S1.** **Log_2_FC and Primers of lncRNAs.** | | | |
| --- | --- | --- | --- |
|  | Log_2_FC | Forward Primer | Reverse Primer |
| LINC02001 | 2.9997 | GAAAAGCGGGCTGCAGATTC | CGCACAACCAGTGCTTACAC |
| MKLN1-AS | 2.3866 | CCGGGCCAATGTCCTATCTC | AAGCGCTTACACCTCAGACC |
| ZBED3-AS1 | 2.1479 | AATGAGGCACTGGAAGACGG | GGTGAACACCAATAAGCGCC |
| LINC01612 | 2.1388 | ACAATACGGGTGAGGAGCAC | TGGTGTAGTTGCTCTCCTGC |
| FLJ22447 | 2.1158 | TGGCACTGTTTTCACTTGCC | CTTCATGGCCCAGGAGCAAA |
| HORMAD2-AS1 | 2.1136 | CCTGGGCCACCTATACAACG | CAAAGCTGTTGAACCTCGGC |
| GRM7-AS3 | 1.9426 | ACTCATGCATGGCACGTCTA | TGGGTTATGTAGCTTCCTGGC |
| LOC105371240 | 1.9422 | AGGCTGGATGTGACATGGTG | AGGTGTGCTGCTTTCACTCA |
| DNMBP-AS1 | 1.9224 | CAGTGGGAATGCCATCGAGA | GCTGGGTGATGACTGAGGTC |
| TALAM1 | 1.8978 | TTCTTCAGACGTGGGTCCCT | AAATACCCTCCCTGGCCCTC |
| TTN-AS1 | 1.8697 | TGCGAAATCCTGTCTTCGGT | CCAGCCTTGACATGCTGGTA |
| UCA1 | 1.8695 | GCACCCTAGACCCGAAACTT | GGGATTACTGGGGTAGGGCT |
| ITGA9-AS1 | 1.8582 | GTGGCATCCCATTTAAAATTGCT | CTCTGCTGCAGGGAGATTACA |
| TP53TG1 | -3.0571 | GAGCTGTCCTAACTCTGCGG | GAGGGTTGGGTACCTTCGTG |
| INO80B-WBP1 | -2.9806 | TCAGTGCTACCGCATCAACC | CACAGCTCTCGAAGGTAGCC |
| LOC102724902 | -2.8354 | TTTGGGTGGAGCCCTTTGAG | GGCTCATATTTACATTCGACACACA |
| IDI2-AS1 | -2.7356 | AAGTTGGCCGTCGAGTGAAG | TGCCTTCACCACTTTCCGAG |
| LOC107985976 | -2.6755 | CACGACCCACCTCAAGTGTT | TCAGAGAAGTACTCTAGCAGACC |
| LOC105379549 | -2.6323 | TGCGATCTCAATTGCCTTTTAGC | TGACACAGAAGAACCCATGAGA |
| GATA2-AS1 | -2.6086 | ACGCCACATGACCTTTCCTT | ACAGTGCCCTTTCGTGATGA |
| LOC105372310 | -2.6031 | AGCAGCACATTGTTCCCAAA | CCTGCAAGCATGACATCAAC |
| LOC101929572 | -2.5362 | GGGGACTAACTCACTGCCAC | TCTATCTGGACAGACGGCCA |
| LOC105370333 | -2.5295 | TGTCATTGAGTCACCTCCGC | ACGCTTTCGAACATGCAGGA |
| LINC01510 | -2.5138 | TTCTGCAAGCCAGTGGGAAC | CAGGGGTGTCTGTCCTCAAA |
| LOC100507412 | -2.5021 | TGGAGAATCTTAACTTTCTTGGTGG | ACGAGAGATGCTCATTTCAACA |
| CTC-338M12.4 | -2.5017 | GGCCCGGTTCTCTTTCACTAA | AGCCAACCATACAGTCAGCC |
| GAPDH |  | ACCCACTCCTCCACCTTTGAC | TGTTGCTGTAGCCAAATTCGTT |
| Log_2_FC, Log_2_fold change. | | | |
