# Supplementary material for: Urinary exosomal long non-coding RNAs as noninvasive biomarkers for diagnosis of bladder cancer by RNA sequencing
Source: Front Oncol. 2022 Sep 1;12:976329. doi: 10.3389/fonc.2022.976329 (PMC9477086; doi:10.3389/fonc.2022.976329)
Supplement: Supplementary file 3 [file Table_2.docx]

| Table S2. The diagnostic performance of lncRNAs in the training cohort. | | | | | |
| --- | --- | --- | --- | --- | --- |
|  | **AUC** | **Sensitivity** | **Specificity** | **PLR** | **NLR** |
| **MKLN1-AS** | 0.773(0.679 to 0.851) | 92 | 52 | 1.92 | 0.15 |
| **TALAM1** | 0.770(0.675 to 0.848) | 96 | 48 | 1.85 | 0.083 |
| **TTN-AS1** | 0.800(0.709 to 0.874) | 94 | 52 | 1.96 | 0.12 |
| **UCA1** | 0.813(0.723 to 0.884) | 92 | 56 | 2.09 | 0.14 |
| AUC, area under the curve. PLR, positive likelihood ratio. NLR, negative likelihood ratio. | | | | | |
