# Supplementary material for: Urinary exosomal long non-coding RNAs as noninvasive biomarkers for diagnosis of bladder cancer by RNA sequencing
Source: Front Oncol. 2022 Sep 1;12:976329. doi: 10.3389/fonc.2022.976329 (PMC9477086; doi:10.3389/fonc.2022.976329)
Supplement: Supplementary file 4 [file Table_3.docx]

| Table S3. The diagnostic performance of lncRNAs in the validation cohort. | | | | | |
| --- | --- | --- | --- | --- | --- |
|  | **AUC** | **Sensitivity** | **Specificity** | **PLR** | **NLR** |
| **MKLN1-AS** | 0.798(0.697 to 0.877) | 79.07 | 67.44 | 2.43 | 0.31 |
| **TALAM1** | 0.752(0.647 to 0.839) | 90.7 | 55.81 | 2.05 | 0.17 |
| **TTN-AS1** | 0.808(0.709 to 0.885) | 76.74 | 76.74 | 3.3 | 0.3 |
| **UCA1** | 0.759(0.655 to 0.845) | 90.7 | 51.16 | 1.86 | 0.18 |
| AUC, area under the curve. PLR, positive likelihood ratio. NLR, negative likelihood ratio. | | | | | |
