# Supplementary material for: Urinary exosomal long non-coding RNAs as noninvasive biomarkers for diagnosis of bladder cancer by RNA sequencing
Source: Front Oncol. 2022 Sep 1;12:976329. doi: 10.3389/fonc.2022.976329 (PMC9477086; doi:10.3389/fonc.2022.976329)
Supplement: Supplementary file 5 [file Table_4.docx]

| **Table S4. The diagnostic performance of the panel.** | | | | | |
| --- | --- | --- | --- | --- | --- |
|  | **AUC** | **Sensitivity** | **Specificity** | **PLR** | **NLR** |
| **Training cohort** | 0.850(0.764-0.913) | 72 | 82 | 4 | 0.34 |
| **Validation cohort** | 0.823(0.726-0.897) | 86.05 | 65.12 | 2.47 | 0.21 |
| **NMIBC** | 0.802(0.730-0.861) | 73.77 | 74.19 | 2.86 | 0.35 |
| **MIBC** | 0.881(0.811-0.932) | 78.12 | 84.95 | 5.19 | 0.26 |
| **Low grade** | 0.768(0.681-0.841) | 95.83 | 50.54 | 1.94 | 0.082 |
| **High grade** | 0.850(0.786-0.901) | 82.61 | 74.19 | 3.20 | 0.23 |
| AUC, area under the curve. PLR, positive likelihood ratio. NLR, negative likelihood ratio. NMIBC, non-muscle-invasive bladder cancer. MIBC, muscle-invasive bladder cancer. | | | | | |
